# Pertraction of Co(II) through Novel Ultrasound Prepared Supported Liquid Membranes Containing D2EHPA. Optimization and Transport Parameters

**DOI:** 10.3390/membranes10120436

**Published:** 2020-12-17

**Authors:** Gerardo León, Asunción María Hidalgo, Beatriz Miguel, María Amelia Guzmán

**Affiliations:** 1 Departamento de Ingeniería Química y Ambiental, Universidad Politécnica de Cartagena, Paseo Alfonso XIII, 30203 Cartagena, Spain; beatriz.miguel@upct.es (B.M.); maguzmanmv@gmail.com (M.A.G.); 2 Departamento de Ingeniería Química, Campus de Espinardo, Universidad de Murcia, 30100 Murcia, Spain; ahidalgo@um.es

**Keywords:** cobalt(II), supported liquid membranes, ultrasound, D2EHPA, counter-transport, transport parameters

## Abstract

Pertraction of Co(II) through novel supported liquid membranes prepared by ultrasound, using bis-2-ethylhexyl phosphoric acid as carrier, sulfuric acid as stripping agent and a counter-transport mechanism, is studied in this paper. Supported liquid membrane characterization through scanning electron microscopy, energy-dispersive X-ray spectroscopy and Fourier transform infrared spectroscopy shows the impregnation of the microporous polymer support by the membrane phase by the action of ultrasound. The effect on the initial flux of Co(II) of different experimental conditions is analyzed to optimize the transport process. At these optimal experimental conditions (feed phase pH 6, 0.5 M sulfuric acid in product phase, carrier concentration 0.65 M in membrane phase and stirring speed of 300 rpm in both phases) supported liquid membrane shows great stability. From the relation between the inverse of Co(II) initial permeability and the inverse of the square of carrier concentration in the membrane phase, in the optimized experimental conditions, the transport resistance due to diffusion through both the aqueous feed boundary layer (3.7576 × 10^4^ s·m^−1^) and the membrane phase (1.1434 × 10^10^ s·m^−1^), the thickness of the aqueous feed boundary layer (4.0206 × 10^−6^ m) and the diffusion coefficient of the Co(II)-carrier in the bulk membrane (4.0490 × 10^−14^ m^2^·s^−1^), have been determined.

## 1. Introduction

Cobalt is associated with many industrial and technological activities such as mining, hydrometallurgy, medicine and the manufacture of batteries, steels, magnetic alloys, catalysts, glass, ceramics, paints, lacquers, etc. [1]. Due to its industrial significance, cobalt production has grown steadily over the last two decades, from 56,635 tonsin 2005 [2] to 124,344 tonsin 2018 [3], leading to both the decrease of primary cobalt resources and the increase in cobaltwaste.

Moreover, the presence of cobalt in the wastewater of the above industries is an important environmental problem because, like other heavy metals, it is not biodegradable and tends to accumulate in living organisms, causing diseases and disorders. The acute effects of cobalt on humans affect cardiovascular, endocrine, hematological, respiratory and nervous systems [4].

All this makes the recovery of cobalt from raw materials and secondary sources very interesting from both environmental and economic reasons.

Different techniques have been described for cobalt removal from aqueous solutions, including flocculation [5], adsorption [6,7,8,9], biosorption [10,11,12], phytoremediation [13], solvent extraction [14,15], ion exchange [16] capacitive deionization [17], electrowinning [18], micellar enhanced ultrafiltration [19], nanofiltration [20], reverse and forward osmosis [21,22], membrane distillation [23], liquid membranes [24,25,26,27] and combined methods [28].

Liquid membranes are receiving great attention as a separation process because they combine the extraction and the recovery processes in a single continuous stage [29]. In a liquid membrane, two miscible phases (feed and product phases) are separated by an immiscible phase (membrane phase). Supported liquid membranes (SLM) are obtained when the pores of a thin microporous solid support are filled with the membrane phase [29].

Traditionally, the filling of these pores has been carried out by impregnation of the microporous support by the liquid membrane solution under pressure or under vacuum. In this paper, we use a novel method based on the effects of ultrasound. The application of ultrasound to a liquid medium causes mechanical vibration and acoustic streaming. As the liquid medium usually contains dissolved gaseous nuclei, ultrasound generates acoustic cavitation (expanding and collapsing them), releasing large amounts of energy that generate, among other effects, shock waves and micro jets [30]. Polyvinylidene fluoride (PVDF) has been selected as the microporous support, due to its greater resistance to the ultrasound mechanical effects [31,32].

Transport in a liquid membrane system is usually improved by adding to the membrane phase a complexing agent (carrier) to carry the diffusing species across the membrane to the product phase [33]. This process can be accompanied by the transport of other chemical species from the product to the feed phase (coupled counter-transport mechanism), which offers the possibility of transporting a component against its own concentration gradient [34].

In this paper we study the Co(II) pertraction from an acetate buffered aqueous feed phase to an aqueous product phase which contains sulfuric acid as stripping agent (protons as counter ions), through an ultrasound prepared supported liquid membrane containing D2EHPA in kerosene, by using a coupled counter-transport mechanism.

To optimize the pertraction process, the effect on Co(II) initial flux of different experimental conditions (pH of the feed phase, carrier concentration in the membrane phase, stripping agent concentration in the product phase and stirring rate in both feed and product phases) is analyzed. From the relation between the inverse of Co(II) initial permeability and the inverse of the square of carrier concentration in the membrane phase, at the optimal experimental conditions, the transport resistance due to diffusion through both the aqueous feed boundary layer and the membrane phase, the thickness of the aqueous feed boundary layer and the diffusion coefficient of the Co(II)-carrier in the bulk membrane phase are determined.

## 2. Theoretical Background

**Figure 1:**
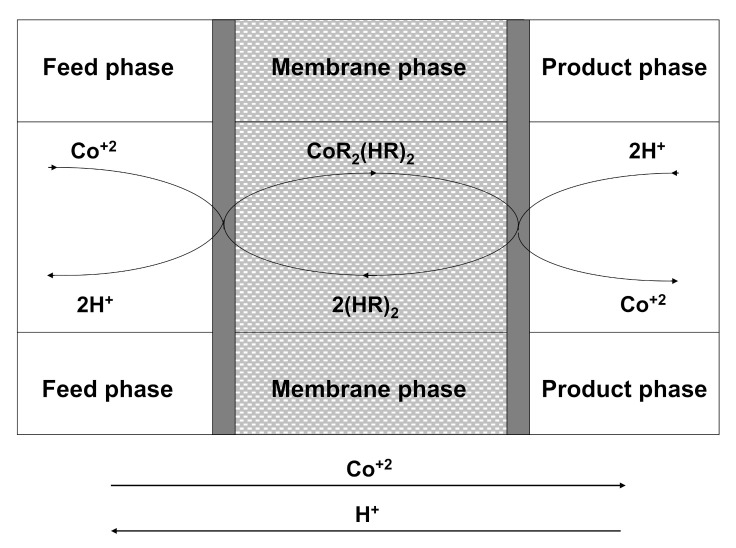
Diagram of the coupled counter-transport of Co(II) ions using D2EHPA as carrier and H^+^ as counter-ion.

The coupled counter-transport of Co(II) ions through a liquid membrane using D2EHPA as carrier and H^+^ as counter ion (sulfuric acid as a stripping agent) is illustrated in Figure 1. Dimerized molecules of carrier (HR)_2_ [35] diffuse from the membrane phase to the feed/membrane interface where they undergo reaction with Co(II). Each Co(II) ion is exchanged for two protons, according to the following equation [36]:

Co^2+^_(aq)_ + 2(HR)_2(org)_ ⇔ CoR_2_(HR)_2 (org)_ + 2H^+^_(aq)_

The Co(II)-carrier complex, CoR_2_(HR)_2_, diffuses through the membrane phase to the membrane/product interface where due to the high acidic conditions of product phase, the described reaction is reversed and protons are exchanged for Co(II) ions, which are released into the product phase, the carrier being regenerated to begin a new separation cycle. A coupled counter-transport mechanism takes place, so that Co(II) and H^+^ travel in opposite directions.

The equilibrium constant of the described reversible reaction (*K_e_*) can be expressed by Equation (1).

(1) $$K_{e}=\frac{{{[\mathrm{CoR}}_{2}{\left(\mathrm{HR}\right)}_{2}]}_{\mathrm{org}}\times {{[H}^{+}]}_{\mathrm{aq}}^{2}}{{{[\mathrm{Co}}^{2+}]}_{\mathrm{aq}}\times {{\left[(\mathrm{HR}\right)}_{2}]}_{\mathrm{org}}^{2}}$$

It can be considered that chemical reactions that take place at the feed/membrane and membrane/product interfaces occur faster than the diffusion processes [37] and the Co(II) transport rate is determined by the rate of diffusion of Co(II) through the feed diffusion layer and the rate of diffusion of the Co(II)-carrier complex through the membrane. The Co(II) flux across the membrane can be obtained by applying Fick’s first diffusion law to the diffusion layer on the feed side (*J_fbl_*) and to the membrane (*J_m_*) through the following equations [38]:

(2) $$J_{fbl}=\frac{{{[\mathrm{Co}}^{2+}]}_{f}-{{[\mathrm{Co}}^{2+}]}_{i,f/m}}{\Delta_{fbl}}$$

(3) $$J_{m}=\frac{{{[\mathrm{CoR}}_{2}{\left(\mathrm{HR}\right)}_{2}]}_{i,f/m}-{{[\mathrm{CoR}}_{2}{\left(\mathrm{HR}\right)}_{2}]}_{i,m/p}}{\Delta_{m}}$$

where Δ*_fbl_* is the transport resistance due to diffusion through the aqueous feed boundary layer (*δ_fbl_/D_aq_*) (s·m^−1^), Δ*_m_* is the transport resistance due to diffusion through the membrane phase(*δ_m_/D_ps_*(s·m^−1^), [Co^2+^]_f_ is the cobalt concentration in the feed phase, [Co^2+^]_i,f/m_ is the cobalt concentration in the feed/membrane interface, [CoR_2_(HR)_2_]_i,f/m_ is the complex concentration in the feed/membrane interface, [CoR_2_(HR)_2_]_i,m/p_ is the complex concentration in the membrane/product interface, *δ_fbl_* is the thickness of the aqueous feed boundary layer (*m*), *D_aq_* is the average aqueous diffusion coefficient of the Co(II) (m^2^·s^−1^), δ_m_ is the thickness of the membrane phase (*m*) and *D_ps_* is the diffusion coefficient of the Co(II)-carrier in the polymeric support.

Due to the different pH values of the feed and the product phases, the distribution coefficient of Co(II) between the membrane phase and the product phase is much lower than that between the feed phase and the membrane phase. Consequently, the concentration of the Co(II)-carrier complex at the membrane/product interface may be considered negligible compared to that at the feed/membrane interface and Equation (3) can be rewritten as

(4) $$J_{m}=\frac{{[{\mathrm{CoR}}_{2}{\left(\mathrm{HR}\right)}_{2}]}_{i,f/m}}{\Delta_{m}}$$

If, as assumed above, chemical reactions are fast compared with the diffusion rate, local equilibrium is reached at the interface, where concentrations are related through Equation (1). Thus, in the steady state, *J_fbl_* = *J_m_*= *J*, and by combining Equations (1), (2) and (4), the following flux expression can be obtained:

(5) $$J=\frac{K_{e}\cdot {{\left[(\mathrm{HR}\right)}_{2}]}^{2}\cdot {{[\mathrm{Co}}^{2+}]}_{f}}{\Delta_{m}\cdot {{[H}^{+}]}^{2}+\Delta_{fbl}\cdot K_{e}\cdot {{\left[(\mathrm{HR}\right)}_{2}]}^{2}}$$

Thus, the permeability coefficient, P = *J*/[Co(II)]_f_, can be written as [38]

(6) $$P=\frac{K_{e}\cdot {{\left[(\mathrm{HR}\right)}_{2}]}^{2}}{\Delta_{m}\cdot {{[H}^{+}]}^{2}+\Delta_{fbl}\cdot K_{e}\cdot {{\left[(\mathrm{HR}\right)}_{2}]}^{2}}$$

From Equation (6), the following expression for 1/P is obtained

(7) $$\frac{1}{P}=\Delta_{fbl}+\frac{\Delta_{m}\cdot {{[H}^{+}]}^{2}}{K_{e}\cdot {{\left[(\mathrm{HR}\right)}_{2}]}^{2}}$$

By plotting 1/P as a function of 1/[(HR)_2_]_2_, at constant pH, a straight line should be obtained with slope (∆*_m_·*[H^+^]^2^)/*K_e_* and ordinate ∆*_fbl_*. Knowing *K_e_* and the pH of the feed solution, ∆*_m_* can be obtained from the slope.

Since ∆*_fbl_ = δ_fbl_/D_aq_*, the value of the thickness of the aqueous feed boundary layer can be calculated if the average aqueous diffusion coefficient is known.

Similarly, since ∆*_m_ = δ_m_/D_ps_*, knowing the thickness of the supported liquid membrane, *D_ps_* can be calculated, while the diffusion coefficient of the Co(II)-carrier in the bulk membrane phase (*D_bm_*) can be obtained by the following equation [39]:

(8) $$D_{bm}=\frac{D_{ps}\cdot \tau^{2}}{\varepsilon }$$

The porosity of the membrane (ε) is usually given by the membrane supplier and the tortuosity of the membrane (τ) can be calculated according to the relationship [40]:

(9) $$\tau =\frac{1+V_{p}}{1-V_{p}}$$

where the volume fraction of polymeric support (*V_p_*) is 1 − ε [41].

## 3. Materials and Methods

### 3.1. Materials

Cobalt(II) chloride (98%), sodium acetate (99%), acetic acid (95%) and sulfuric acid (95–98%) were purchased from Panreac. Bis-2-ethylhexyl phosphoric acid (97%) was obtained from Sigma-Aldrich, Madrid, Spain. Kerosene (99%) was supplied by BDH Middle East, Dubai, UAE. A microporous hydrophobic PVDF ultrafiltration membrane (Millipore Durapore GVHP 10, Merck, Madrid, Spain), was utilized as support for the liquid membrane (geometrical area 20 cm^2^, porosity of 75%, pore size of 0.22 μm and thickness of 125 μm).

### 3.2. Methods

#### 3.2.1. Preparation of Supported Liquid Membrane

**Figure 2:**
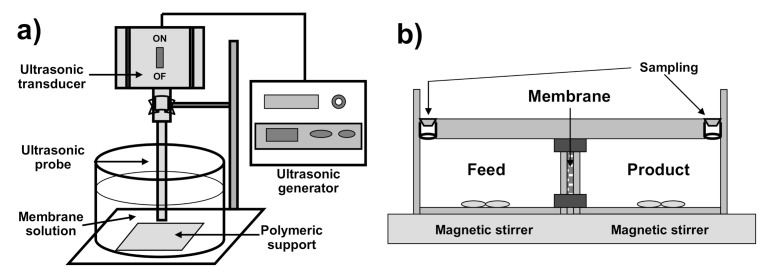
Schematic representations of: (**a**) sonication system; (**b**) experimental transport cell.

The liquid membrane phase was constituted by kerosene solutions of D2EHPA at concentrations between 0.2 and 0.8 M. The pores of the microporous support were filled with the membrane solution by applying ultrasound, using Labsonic M (Sartorius SA, Madrid, Spain) ultrasound equipment (titanium probe 10 mm diameter, sound rating density 130 W/cm^2^), at 30 KHz, 150 μm, for 30 min (three times for 10 min, with 5 min intervals between them), and the active layer of the polymeric support at a distance of 16 mm from the ultrasound probe [24] (Figure 2a).

#### 3.2.2. Supported Liquid Membrane Characterization

Scanning electron microscopy (SEM), energy-dispersive X-ray spectroscopy (EDX) and infrared spectrometry (IR) were used to study the impregnation of the pristine PVDF porous polymeric support with the liquid membrane phase by the effect of ultrasound (PVDF-USLM).

The outer surface and elemental composition of both PVDF and PVDF-USLM were analyzed by SEM using a HITACHI S-3500N apparatus, containing secondary and backscattered electron detectors (Hitachi High-Technologies Corporation, Tokyo, Japan), equipped with an EDX XFlash 5010 analysis system (Brukers AXS, Karlsruyhe, Germany). 15 kV, 10 mm work distance, samples sputtered with a thin layer of platinum during 90 s by a sputter coater Polaron SC 7640 (Quorum Technologies, Newhaven, UK) and 5000× magnification were used in SEM study, while 15 kV and 15 mm work distance were used in EDX analysis.

The outer surface chemical functional groups of both PVDF and PVDF-USLM were analyzed by using a NICOLET 5700 FTIR equipment (ThermoFischer Scientific, Waltham, MA, USA), in transmittance mode from 400 cm^−1^ to 4000 cm^−1^.

#### 3.2.3. Transport Experiments

Transport studies were carried out using a permeation cell consisting of two identical compartments, containing 250 cm^3^ (V) of feed or product phase, separated by the supported liquid membrane with an effective area (A) of 15 cm^2^ [24] (Figure 2b). As feed phase, aqueous solutions of Co(II) between 0.010 M to 0.200 M (10 mol/m^3^ to 200 mol/m^3^) in 0.2 M acetate buffer, with pH ranging from 3 to 7, were used, and aqueous sulfuric acid solutions between 0.005 and 1 M were used as product phase. Both phases were mechanically stirred at speeds ranging from 50 to 400 rpm, at room temperature.

#### 3.2.4. Analytical Methods and Calculations

Samples from the product phase compartment were taken every 30 min and Co(II) concentrations were determined by flame atomic absorption spectrophotometry at 240.7 nm usinga Shimadzu AA-2600 apparatus (Duisburg, Germany). The experiments were carried out in duplicate and the results obtained showed less than 3% deviation.

Initial Co(II) fluxes were determined, according to Equation (10) [42], from the slope of the straight line obtained when plotting the Co(II) concentration in the product phase ([Co^+2^]_pt_) as a function of time during the first four hours of the experiment, because of the linear relationship observed during that time.

(10) $$J=\frac{V}{A}\frac{d{\left[{\mathrm{Co}}^{2+}\right]}_{\mathrm{pt}}}{\mathrm{dt}}$$

Initial Co(II) permeability values (P) were determined, according to Equation (11) [43], from the straight line obtained when plotting ln[*C_0_*/(*C_0_* − *C_pt_*)] versus time during the first four hours of the experiment, when a linear relationship was observed

(11) $$\ln\frac{{\left[{\mathrm{Co}}^{2+}\right]}_{f0}}{{\left[{\mathrm{Co}}^{2+}\right]}_{f0}-{\left[{\mathrm{Co}}^{2+}\right]}_{\mathrm{pt}}}=\frac{A}{V}\;P\;t$$

where [Co^2+^]_f0_ is the initial Co(II) concentration in the feed phase.

The instability of the supported liquid membrane was determined from the decrease in Co(II) flux through the membrane in four successive experiments using the same membrane at the optimal experimental conditions.

## 4. Results

### 4.1. Membrane Characterization

**Figure 3:**
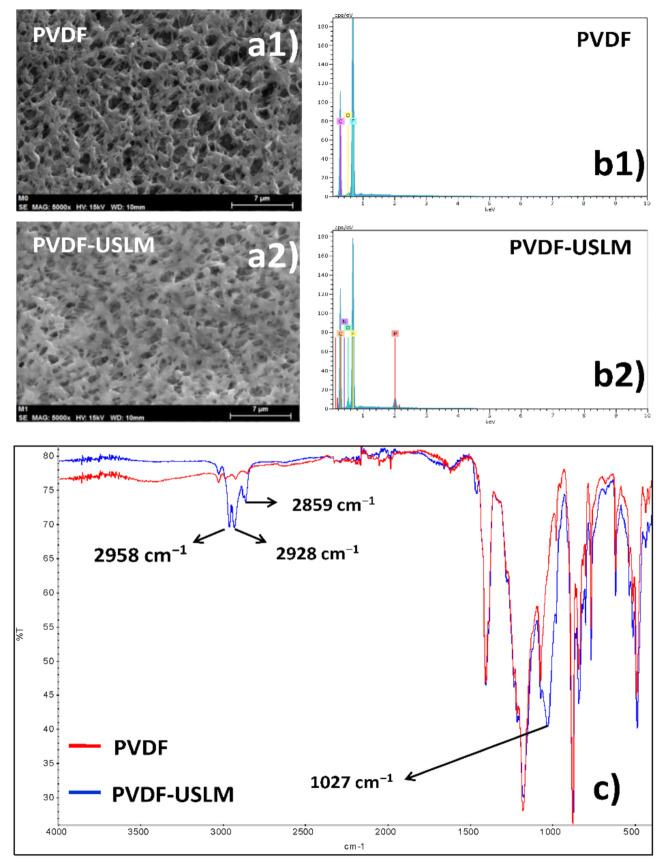
Membrane characterization of PVDF and PVDF-USLM by (**a1**,**a2**) SEM; (**b1**,**b2**) EDX; (**c**) FTIR.

Figure 3 shows SEM, EDX and FTIR of both PVDF and PVDF-USLM.

SEM shows that the surface microstructure, in terms of the surface morphology and porous structure, was not significantly changed after sonification/impregnation process, though the surface roughness of the impregnated support slightly decreased, which must be a consequence of filling the pores with the liquid membrane phase.

EDX characterization results of the shell surface shows the absence of phosphorus in the original PVDF support but its presence in the PVDF-USLM membrane. This confirms that the polymeric porous support has been adequately impregnated with the liquid membrane phase through the application of ultrasound.

The analysis of the FTIR of PVDF-USLM film shows the presence of several significant bands which are not present in PVDF film. The bands between 2958 and 2859 cm^−1^ (corresponding to C-H stretching) show the presence of methyl and ethyl groups and the band at 1027 cm^−1^ (corresponding to P–O–C stretching) shows the presence of P–O–CH_2_– groups. This supports the incorporation of the liquid membrane phase (D2EHPA in kerosene) into the PVDF microporous support during the sonification process with the liquid membrane phase.

### 4.2. Optimization of Co(II) Transport Process

**Figure 4:**
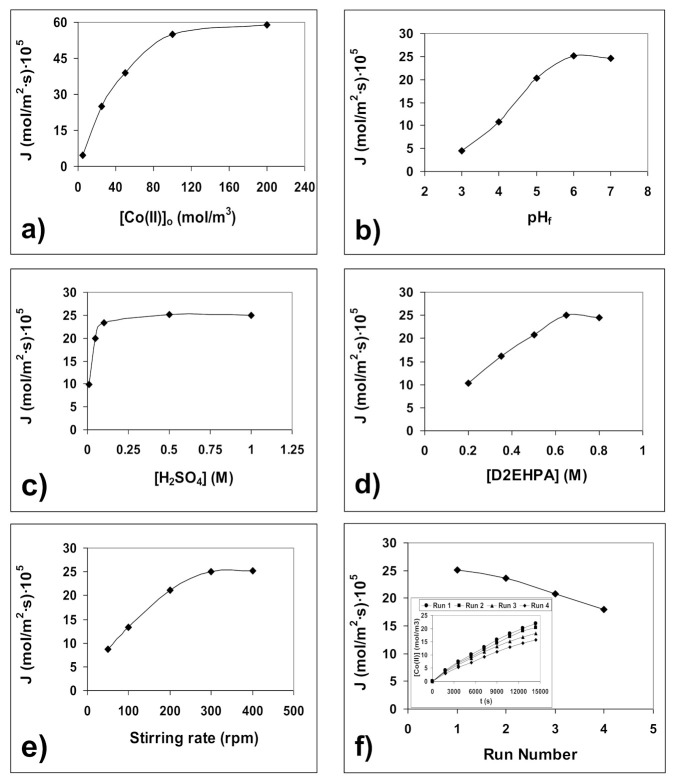
Influence on Co(II) flux of: (**a**) initial feed pH; (**b**) sulfuric acid concentration in product phase; (**c**) carrier concentration in membrane phase; (**d**) stirring rate in both aqueous and product phases; (**e**) initial Co(II) concentration in feed phase; (**f**) successive runs with the same membrane.

The influence on the Co(II) pertraction (expressed in terms of flux) of different parameters such as feed phase Co(II) concentration and pH, sulfuric acid concentration in the product phase, carrier concentration in the membrane phase and stirring speed in both feed and product phases, is shown in Figure 4a–f.

Figure 4a shows the effect of Co(II) concentration in the feed phase on Co(II) flux. The flux increased as the Co(II) concentration in the feed phase increased from 10 mol/m^3^ to 100 mol/m^3^ due to the presence of a higher number of Co(II) ions in the feed/membrane interface, which facilitated the formation of Co(II)-carrier complex leading to a higher transport. A further increase in Co(II) concentration has no significant effect on flux due to saturation of the feed/membrane interface by the Co(II) ions. A Co(II) concentration of 0.025 M (25 mol/m^3^) was selected for subsequent experiments as it is the lowest concentration at which significant flux variations were observed with the other parameters studied.

As shown in Figure 4b, the flux increased when feed pH increased between pH 3 and pH 6 and then remained constant. At low feed pH (high [H^+^]), the equilibrium of the extraction reaction was highly displaced to the left, and no Co(II)-carrier complex was formed. Moreover, the low proton gradient between product and feed phases generated a low driving force. As the feed pH increased ([H^+^] decrease), both the equilibrium of the extraction reaction shifted towards the right (more Co(II) carrier complex is formed) and the proton gradient between the product and the feed phases increased. Consequently, Co(II) transport from the feed to the permeate phase increased. Above pH 6, the OH^-^ competes with the carrier to form a Co(II) complex and so its transport decreases. Thus, a pH of 6 in the feed phase was maintained throughout the study.

The pertraction of Co(II) from aqueous feed phase across the membrane phase is dependent on the concentration of the stripping agent (H_2_SO_4_) present in the product phase (Figure 4c). The results show that the Co(II) flux increased sharply as the sulfuric acid concentrations raised from 0.005 to 0.100 M, and then more slowly up to 0.5 M. At higher sulfuric acid concentrations, the Co(II) flux remained practically constant. These results confirm that the presence of a proton gradient between the product and the feed phases is essential for a high mass transfer. Therefore, a sulfuric acid concentration of 0.5 M in the permeate phase was chosen for further experiments.

The effect of the carrier concentration in the membrane phase on Co(II) flux is shown in Figure 4d. As can be seen, Co(II) flux increased as the carrier concentration increased from 0.2 to 0.65 M, but further increases in carrier concentration had no significant effect on Co(II) flux. According to the equilibrium of the extraction reaction (reaction 1), the higher the carrier concentration in the membrane phase, the more Co(II)-carrier complex is formed. Above 0.65 M, both the saturation of the feed/membrane interface by the carrier and the higher viscosity of the membrane phase led to the Co(II) flux remaining constant. Therefore, a carrier concentration of 0.65 M was used in subsequent experiments.

The effect of stirring rate on Co(II) flux is shown in Figure 4e. The flux increased as the stirring rate increased from 50 to 300 rpm, above which no appreciable variation was observed. This indicates that the boundary layers thickness diminished continuously as the stirring rate increased and that minimum values of these boundary layers (minimal diffusion resistance due to the boundary layers) are reached at 300 rpm and above. Therefore, further experiments were carried out at 300 rpm.

The instability of the supported liquid membrane, measured as the decrease in Co(II) flux in four successive runs, is shown in Figure 4f, where the variation of Co(II) concentration with time in those four successive experiments with the same membraneis also shown. The flux in runs 2, 3 and 4, expressed as percentage with respect to the flux in run 1, were 94%, 83% and 71%, respectively. These flux decreases are lower than those found by other authors using similar supported liquid membranes, but prepared by immersion of the polymeric support in the liquid membrane phase [44].

### 4.3. Determination of Transport Parameters

**Figure 5:**
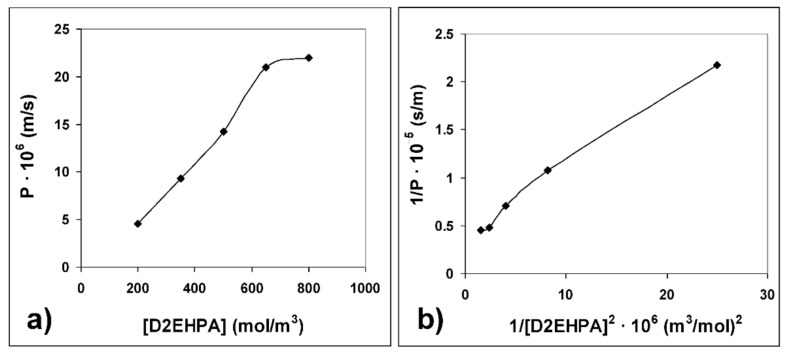
(**a**) Effect of the carrier concentration in the membrane phase on Co(II) initial permeability; (**b**) inverse of Co(II) initial permeability versus the inverse of the square of carrier concentration in the membrane phase.

The effect of the carrier concentration in the membrane phase on Co(II) initial permeability is shown in Figure 5a. As in the case of initial flux, initial permeability increased as the carrier concentration increased from 200 to 650 mol·m^−3^, but further increases in carrier concentration had no significant effect on Co(II) permeability.

From data obtained when plotting the inverse of Co(II) initial permeability versus the inverse of the square of carrier concentration in the membrane phase (Figure 5b, R^2^ = 0.9908), at constant pH (pH = 6), knowing the value of *K_e_* of D2EHPA (*K_e_* = 1.1 × 10^−7^ [45]) and using Equation (7), the transport resistance due to diffusion through the aqueous feed boundary layer (*∆_fbl_*) and due to diffusion through the membrane (*∆_m_*) were calculated (Table 1). From these values and data for the membrane polymeric support provided by the supplier (*δ_m_*), the *D_aq_* value of 1.07 × 10^−10^ m^2^·s^−1^ [46] and the Equations (8) and (9), the thickness of the aqueous feed boundary layer (*δ_fbl_*) and the diffusion coefficient of the Co(II)-carrier complex in the bulk membrane phase (*D_bm_*) were obtained (Table 1).

## 5. Conclusions

This paper has analyzed the optimization and the determination of transport parameters of the Co(II) pertraction through novel ultrasound prepared supported liquid membranes by using a coupled counter-transport mechanism, with D2EHPA as carrier in the membrane phase and sulfuric acid as stripping agent (protons as counter-ions) in the product phase. SEM, EDX and FTIR characterization of the supported liquid membrane show good impregnation of the microporous polymer support by the membrane phase throughthe action of ultrasound. To optimize the pertraction process, the effect of different experimental conditions on Co(II) initial fluxes was studied. The optimal experimental conditions were: feed phase pH 6, 0.5 M sulfuric acid in product phase, carrier concentration 0.65 M in membrane phase and stirring speed of 300 rpm in both phases. Supported liquid membrane shows great stability (71%) after four successive runs (four hours each run). From the relation between the inverse of Co(II) initial permeability and the inverse of the square of carrier concentration in the membrane phase, in the optimized conditions, transport resistance due to diffusion through the aqueous feed boundary layer (*∆_fbl_*) and transport resistance due to diffusion through the membrane (*∆_m_*) were calculated as being 3.7576 × 10^4^ sm^−1^ and 1.1434 × 10^10^ sm^−1^, respectively. The thickness of the aqueous feed boundary layer (*δ_fbl_*) was 4.0206 × 10^−6^ m and the membrane diffusion coefficient of the Co(II)-carrier complex through the membrane (*D_bm_*) was 4.0490 × 10^−14^ m^2^·s^−1^.

## Figures and Tables

**Table 1:** Transport parameters of Co(II) pertraction through ultrasound prepared supported liquid membranes containing D2EHPA as carrier.

∆*_fbl_* (sm^−1^)	∆*_m_* (sm^−1^)	δ*_fbl_* (m)	*D_bm_* (m^2^·s^−1^)
3.7576 × 10^4^	1.1434 × 10^10^	4.0206 × 10^−6^	4.0490 × 10^−14^
